# Hypogonadotropic hypogonadism in men with hereditary hemochromatosis

**DOI:** 10.1186/s12610-017-0057-8

**Published:** 2017-07-08

**Authors:** Rabih El Osta, Nicolas Grandpre, Nicolas Monnin, Jacques Hubert, Isabelle Koscinski

**Affiliations:** 1 Urology Department of Brabois University Hospital, CHU Nancy, Vandœuvre-lès-Nancy, France; 2 Hematology department, CHRU Strasbourg, Strasbourg, France; 3 Laboratoire de Biologie de la Reproduction, CECOS Lorraine, CHU Nancy, Nancy, France

**Keywords:** Male infertility, Hypogonadotropic Hypogonadism, Hereditary Hemochromatosis, infertilité masculine, hypogonadisme hypogonadotrope, Hémochromatose Héréditaire

## Abstract

Hereditary hemochromatosis is a genetic disease that progresses silently. This disease is often diagnosed late when complications appear. Hypogonadotropic hypogonadism (HH) is one of the classical complications of hemochromatosis. Its frequency is declining probably because of earlier diagnosis and better informed physicians. Certain symptoms linked to HH can have an impact on a patient’s sexuality, such as decreased libido, erectile dysfunction, and impairment of ejaculation, as well as on his reproductive capacities.

This review is based on an online search in English, French and German language publications found in PubMed/Medline, up to 23 September 2016 using the following key word: Male infertility, Hypogonadotropic Hypogonadism, Hereditary Hemochromatosis.

Thirty-four papers met these inclusion criteria. This review describes the impact of iron overload on male fertility, resulting in hypogonadotropic hypogonadism and proposes treatment modalities.

## Résumé

L’Hémochromatose Héréditaire est. une maladie génétique qui évolue en silence. Son diagnostic est. souvent fait tardivement, au stade des complications. Même si son incidence diminue, l’Hypogonadisme Hypogonadotrope (HH) est. l’une des complications classiques de l’Hémochromatose. Ceci est. probablement le résultat d’un diagnostic plus précoce, d’une meilleure information des médecins. Certains symptômes en lien avec l’HH, peuvent avoir un impact sur la sexualité (diminution de la libido, dysfonction érectile ou troubles de l’éjaculation…) ainsi que sur la reproduction.

Cette revue repose sur une recherche online en langue anglaise, française et allemande de publications disponibles sur PubMed/Medline, jusqu’au 23 sept. 2016 à partir des mots clés suivants: infertilité masculine, hypogonadisme hypogonadotrope, Hémochromatose Héréditaire. Trente quatre publications ont satisfait aux critères de sélection. Cet article de revue décrit l’impact d’une surcharge en fer sur la fertilité masculine, notamment via l’Hypogonadisme Hypogonadotrope qu’elle induit et propose des modalités de traitement.

## Background

Hemochromatosis is an iron overload disease characterized by normal erythropoiesis, an increase in the saturation coefficient of transferrin (≥ 45%), an increase in the concentration of serum ferritin (≥300 μg/L in a human) and a parenchymal iron deposition caused by low levels of hepcidin [1]. It is the most common inherited disease in France with an estimated prevalence of 0.3% [2]. Due to its frequency, and its potentially complications, hemochromatosis has become nowadays a public health problem [3].

Iron overload causes endocrine dysfunction, particularly on the pituitary axis [4], with a potential impact on fertility. Indeed, iron overload can affect fertility through diverse mechanisms like HH, diabetes and cirrhosis. This review focuses on the reproductive dysfunction associated with iron overload-induced HH and proposes a patient management strategy which preserves the fertility of affected patients.

## Materials and methods

Key words including ‘hemochromatosis’, ‘iron’, ‘male fertility’, ‘spermatogenesis’, and ‘hypogonadism’ were explored in PubMed and articles in English, French and German were included (up to 23 September 2016). The titles and abstracts of the resulting articles were evaluated by the second first author and last authors (REO, NG, and IK). Inclusion criteria were articles that focused on current knowledge in hemochromatosis, iron metabolism and human male fertility. In addition, some background articles on reproductive complications of endocrinopathies were included. Case reports which illustrated interesting elements of the disorder were also included.

## Iron metabolism

The total amount of iron in the organism is approximately 4 g for men and 3 g for women [5]. These 4 g of iron are bound to different proteins like hemoglobin or transferrin. Iron is a constituent of the respiratory chain and cytochromes; it plays the role of enzymatic cofactor, and is implicated in DNA replication or mRNA transcription [6].

Almost all iron is recycled. Digestive absorption balances out physiological loss, which is significant for women contrary to men because of menstruation. Fe^2+^ is transported from the intestinal lumen into the cytoplasm of the epithelial duodenal and jejune cells by the transporter Divalent Metal Transporter 1 (DMT1). Here, iron can be stored as ferritin or can be transported through the basal epithelial membrane by ferroportin and be exported into the lymphatic system. Iron is distributed to target tissues via transferrin which is synthetized by the liver. The iron-transferrin complex binds to the ubiquitous Transferrin Receptor (TfR-) 1 and to TfR-2, which is mainly expressed in the liver. One of the regulation mechanisms of the seric transfer of iron involves the peptide hormone hepcidin, a hormone synthezised in the liver. In physiological conditions, hepcidin synthesis is regulated by some proteins expressed in hepatocytes: Hereditary hemochromatosis Protein (HFE), transferring receptor 2 (TfR-2), hemojuvelin and transferrin. Hepcidin expression is also regulated by pathologic processes such as hypoxy or inflammation [6, 7].

HFE-TfR-1 complexes on the surface of hepatocytes sense the saturation of iron-bound transferrin in the serum. At low transferrin saturation, HFE is sequestered by TfR-1. As serum iron saturation increases, HFE is dislodged from its overlapping binding site on TfR-1 by the complex iron-transferrin. HFE is then free to interact with TfR-2 and signal the up-regulation of hepcidin. Increased levels of circulating hepcidin lead to a reduction in intestinal iron absorption and macrophage iron release, possibly through interaction with iron-export proteins such as ferroportin [8]. This binding allows the internalization of the ferroportin-hepcidin complex. In healthy humans, the peptide hormone hepcidin plays a key role in iron homeostasis like insulin in glucose metabolism [9]. If either HFE is mutated or absent, the complex is unable to sense increased serum transferring saturation, and dysregulation or iron homeostasis occurs. The most frequent mutation of *HFE* is C282Y [3].

## Iron and spermatogenesis

Spermatogenesis requires iron both for the synthesis of DNA and for germ cell growth. In addition, spermatids and mature spermatozoa are very sensitive to oxidative stress [10]. Thus, some evidence indicates that iron supply to germ cells may be tightly regulated by the blood testis barrier [11]. Within the seminiferous tubules, spermatogonia and spermatocytes I acquire loaded ferritin iron from the nearby Sertoli cells. At the spermatid stage, iron is discharged to Sertoli cells which redistribute it to a new generation of spermatocytes. During spermatogenic development, iron is carried along from primary spermatocytes to spermatids; and from spermatids iron is recycled to the apical compartment of Sertoli cells, which traffic it back to a new generation of spermatocytes [11, 12].

**Fig. 1:**
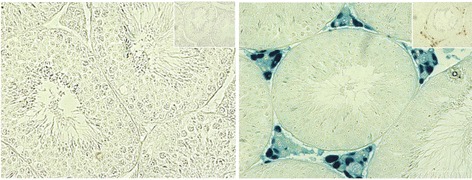
Testis Histopathology. Perl’s staining highlights iron in the interstitium and in the basal membrane of seminiferous tubules in case of iron overload (From [17])

**Fig. 2:**
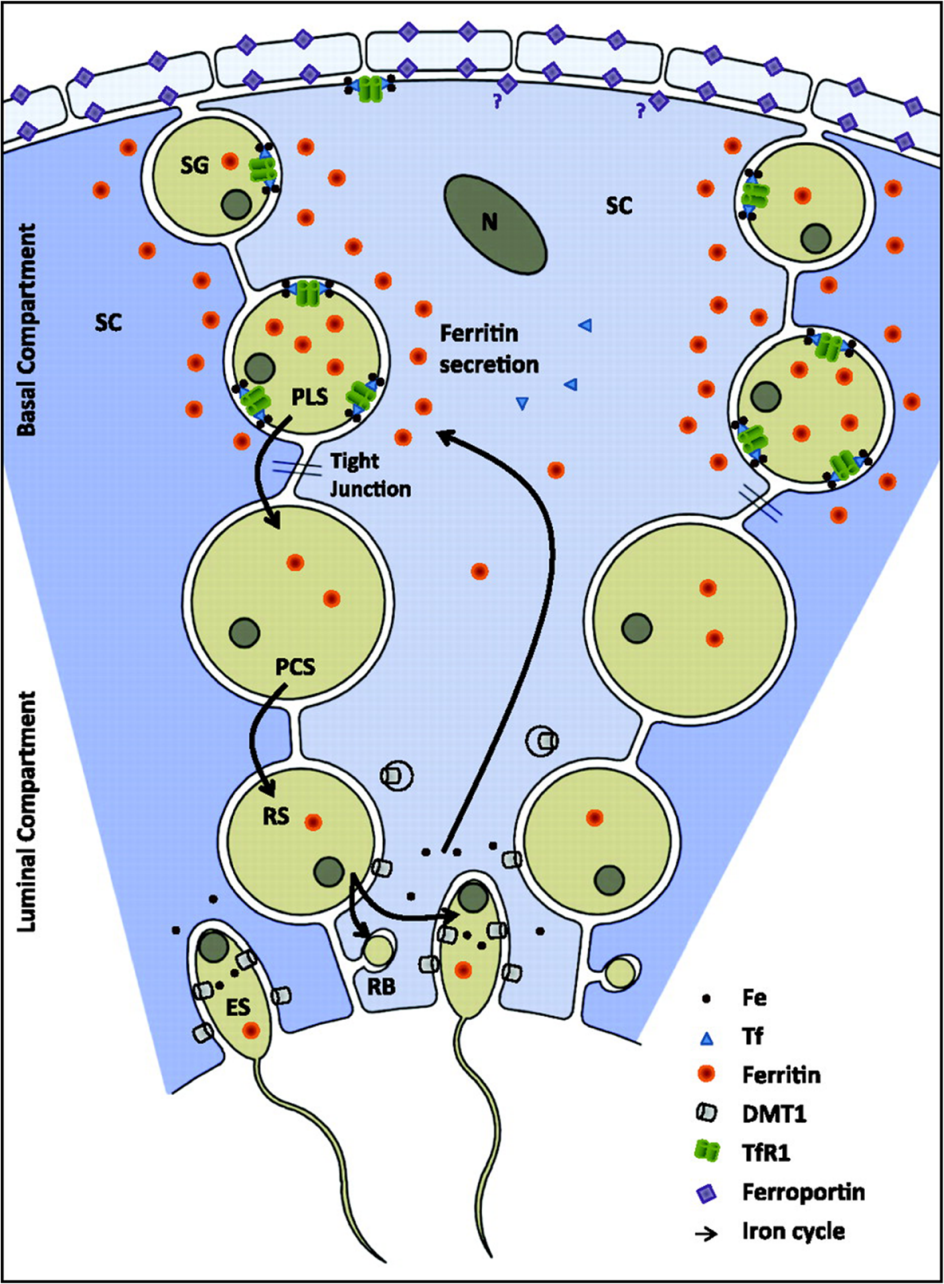
Model for compartmentalized iron transport in the testis. The seminiferous tubule (SFT) is partially protected from systemic iron overload. Here is a model of testis showing the anatomy of interstitial tissue and SFT, where male germ cell development proceeds from the SFT basal membrane (BM) to the SFT lumen (Lu). SC, Sertoli cell; SG, spermatogonia; PLS, preleptotene spermatocyte; PCS, pachytene spermatocyte; RS, round spermatid; ES, elongated spermatid. Three SC and their immediate surroundings are shown. Iron transport across the SFT basal membrane is very limited. Within the SFT some SG and mainly primary spermatocytes acquire iron-loaded ferritin from SC, and upon maturation elongating spermatids return iron to the SC, which traffic it back to a new generation of spermatocytes. Obligatory iron losses through spermatozoa that leave the testis are replenished by the peripheral circulation through the transferrin-TfR-1 system. Ferroportin likely plays its main role in iron trafficking across the interstitial space, where selective barriers at the smooth muscle cells of blood vessels and the peritubular myoid cells provide the male germ cells with additional protection from the periphery. RB, residual body. (From [17])

A study of a mouse model carried out in 2012 highlighted the importance of the basal membrane of the seminiferous tubules as a protective barrier [11]. Indeed, in case of iron overload, a large accumulation of iron is found in peripheral tissues and in the interstitial tissue of the testis and a much smaller iron accumulation can be found in the seminiferous tubules (see Fig. 1). Furthermore, this study highlights the recycling of iron within the seminiferous tubules (see Fig. 2) [11].

The transport of iron through the basal membrane of the seminiferous tubules is limited and indicates a largely autonomous iron cycle to provide effective protection against body overload.

It is possible to differentiate the direct effects of iron overload on fertility such as hypogonadotropic hypogonadism (HH) from the indirect damage it causes, such as diabetes or liver cirrhosis. This review focuses on HH.

## Hypogonadotropic hypogonadism in iron overload

### Epidemiology

In iron overload, hypogonadism is the second most common endocrine abnormality after diabetes. Its frequency in the literature ranges from 10 to 100% [4]. Indeed, older studies reported cases of patients at an advanced stage of hemochromatosis [13]. Hypogonadism was reported in 6.4% of patients with hemochromatosis in the largest cohort [4]. The study by Mc Dermott et al. that followed up 191 patients with hemochromatosis over 20 years, reported hypogonadism in 89% of patients with liver cirrhosis and 33% in patients with diabetes, both representing a complication of iron overload [4]. In that study, the rate of hypogonadism decreased between the beginning (14.6%) and the end of the study (3%) [4], suggesting a potential influence of early detection and management of iron overload as potential disease modifiers of the natural history of the disease.

### Physiopathology

According to McDermott and Walsh, hypogonadism occurs at a relatively advanced stage of iron overload. Even if hypogonadism is generally diagnosed in patients with known iron overload, it could in some cases represent the main reason to consult a physician with a secondary diagnosis of iron overload [14]. Due to social changes in the reasons for consulting a physician, notably the desire for children in men above 50, and the epidemiological landscape of iron overload-related complications in the elderly, hypogonadism could be more frequently diagnosed in Reproduction facilities.

Since the first description by Althausen and Kerr in 1933 [15], endocrine disorders related to iron-overload are better recognized [15, 16]. Some authors have suggested a testicular origin of hypogonadism [11, 17] but a pituitary origin is more commonly accepted [13]; however, regarding the hypothesis of a gonadic origin of hypogonadism, Lucesoli et al. reported in vivo experiments on rat testis response to acute iron overload: histopathology showed an iron deposition in the interstitial tissue at the periphery of seminiferous tubules and a rarefication of germ cells in them [17]. Authors also highlighted an association between a moderate iron increase in the testis and an increase in oxidative stress in the testis with oxidative damage to lipids, proteins, and DNA. These observations confirmed the importance of the blood-testis barrier in the protection of germ cells against iron overload [17, 18]. In addition, the study by Lucesoli et al. emphasized the possibility of testicular damage directly related to iron deposition. Indeed, the iron deposition found in the testis suggested the potential involvement of testicular participation in the pathogenesis of hypogonadism associated with iron overload. These animal experimentations are in concordance with the case reported by Vogt et al. [19]. They reported a case of a 45-year-old man presenting with hemochromatosis diagnosed 4 years earlier and consulting for a decreased libido and an erectile dysfunction suggesting hypogonadism. Gonadotrophin and testosterone levels were lower. The response of Leydig cells to human Chorionic Gonadotropin ﻿(hCG) injections was abnormally weak. The testicular histology was abnormal: maturation arrest at the spermatocyte stage was associated with an iron deposit in endothelial cells and perivascular spaces. Sertoli cells and Leydig cells contained no siderosomes but some lipofuscin, which is a pigment resulting from cell debris related to lysosomes aging in cases of cell sufferance [19]. However these testicular deposits appeared to be variable [20–22].

Concerning the hypothesis of a pituitary origin of HH, the literature suggests that only the gonadotropic axis is disturbed. A review provided in 1996 reported hypogonadism in 46% of cases and only a subclinical deficiency of other axes (15% somatotropic, 8% lactotropic, 4% thyreotropic and 1.5% corticotropic) [23]. The mechanism seemed to be the same whatever the etiology of the hemochromatosis (congenital or secondary). An iron deposit in gonadotropic cells of the anterior part of pituitary led to a decreased production of Follicle Stimulating Hormone (FSH) and Luteinizing Hormone (LH) by these cells [24].

### Treatment modalities

In 1997, Oehninger et al. illustrated the unusual presentation of infertility linked to HH and due to retrograde ejaculation in a 44-year-old patient with hemochromatosis (homozygosity *C282Y*). They also showed that treatment with gonadotropin (hCG 2500 U twice a week and FSH 75 IU three times per week) associated with phlebotomy allows an improvement in the fertility of these patients [25] while dietary precautions, chelator administration and phlebotomy alone were not sufficient.

Another treatment consisted of testosterone hormonal replacement therapy in combination with phlebotomy. Indeed, patients with hemochromatosis have chronic liver disease and low testosterone blood levels with repercussions on erectile function and libido. Kley et al. showed the benefit of treatment with testosterone enanthate on the quality of life, libido and erectile function [26]. These results agreed with the publication of Gama et al. which presented the case of a 44-years-old man with hemochromatosis C282Y/C282Y and suffering from HH [27]. A replacement therapy with testosterone esters (Sustanon® then Proviron®) was administered for 6 years associated with regular phlebotomy (once a week for 4 years). The patient’s condition improved considerably. The substitution was stopped after 6 years. A complete restoration of pituitary function was obtained after treatment by the association of phlebotomy and hormonal replacement therapy [27]. In 2001, Hamer et al. confirmed this result with another case [28]. This patient was 36 years old, and presented also the homozygous mutation of the hemochromatosis allele C282Y/C282Y. He had been diagnosed at the stage of a prefibrotic liver without cirrhosis associated with HH. The history revealed 2 years of erectile dysfunction, decreased libido and infertility. Physical examination revealed symptoms of hypogonadism (a decrease of androgen-dependent hairiness, testicular atrophy and azoospermia). Because of the couple’s desire for a child, the initial phlebotomy (every 14 days, volume not indicated in the publication) was increased to a total of 700 mL per week. Testosterone intake was likewise increased from 100 mg every 3 weeks to 250 mg every 3 weeks. A year after the start of replacement therapy, the patient’s wife was pregnant and the patient’s sexual disorders vanished. After a few months, phlebotomy was carried out again only once a month. The replacement therapy was continued for another year. A yearly control showed no disorder related to hypogonadism and sexual behavior. The patient continued phlebotomy but no longer required replacement therapy. Thus, although testosterone was necessary for a rapid correction of sexual problems, only intensive phlebotomies allowed the normalization of endocrine function [28]. Moreover, the age of the patient on diagnosis also appeared to be key in the reversibility of gonadotropic disorders: in another study, Cundy et al. reported the follow-up of six men suffering from hemochromatosis and treated by venesection therapy: only the youngest patient under 40 presented a complete reversal of his hypogonadotropic hypogonadism [29].

## Discussion

Firstly, regarding pituitary damage, the literature data suggest that it concerns only the gonadal axis. This may result from the exclusive iron deposition in the gonadotropic cells of the anterior pituitary gland leading to a defect of FSH and LH production that explains hypogonadism [24].

Secondly, hypogonadism in hemochromatosis has been long considered irreversible [30–33]. However, some examples of secondary hypogonadism in hemochromatosis with a recovered endocrine function prove that this is not always the case. The combination of iron depletion and hormonal replacement therapy appears to be the best strategy for a rapid improvement in a patient’s health status and quality of life. Replacement therapy helps to maintain a physiological level of testosterone and its metabolites (including dihydrotestosterone and estradiol) to optimize the maintenance of libido and sexual function [34]. In addition, the correction of iron overload by phlebotomy is not always sufficient to normalize pituitary function [29].

**Fig. 3:**
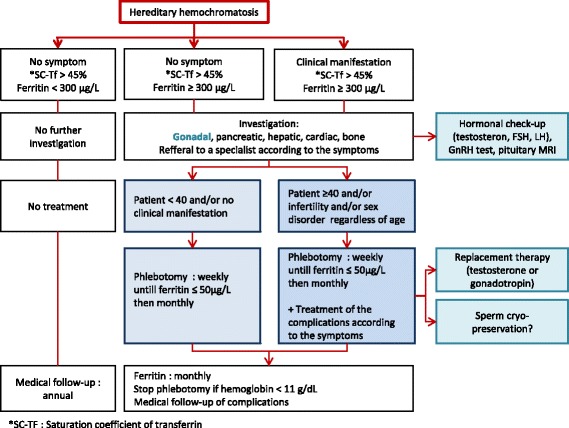
Management optimisation of male patients with hereditary hemochromatosis. In patients presenting increased Ferritin ≥300 μg/L, with or without clinical manifestations, investigations should be provided including a hormonal check-up. In case of abnormal sex hormone concentrations, phlebotomy weekly should be proposed alone if patients are younger than 40, systematically associated with gonadotrophin treatment if 40 or older. According to cases reported in the literature, we also recommend a systematic replacement therapy with gonadotropin associated with phlebotomy in infertile hereditary hemochromatosis patients until they obtained the desired child. Finally, before stopping the replacement therapy and returning to a less intense rhythm of phlebotomy, cryopreservation of spermatozoa is offered to them

These data emphasize that the age at diagnosis and the stage of the disease - especially liver complications - are determining factors for the success of phlebotomy, perhaps because of other indirect effects of Idiopathic Hereditary Hemochromatosis (IHH) (e.g. diabetes and cirrhosis). It is crucial to establish the diagnosis and to start treatment at an early stage of the disease. Thus, patients diagnosed for hereditary hemochromatosis and suffering from infertility or other reproductive disorders should have a brain Magnetic Resonance Imaging (MRI) and a Gonadotropin Releasing Hormone (GnRH) test in order to detect pituitary iron deposits. They could thus be treated by a combined therapy of hCG and phlebotomy. Figure 3 summarizes a suggestion of management optimization of a male patient with hereditary hemochromatosis including a hormonal check-up once Ferritin is ≥300 μg/L, whatever the clinical manifestation of the patient. In case of abnormal sex hormone concentrations, phlebotomy weekly should be proposed alone if patients are younger than 40, systematically associated with gonadotrophin treatment if 40 or older. According to cases reported in the literature, we recommend a systematic replacement therapy with gonadotropin associated with phlebotomy in hereditary hemochromatosis patients with infertility until they obtain the desired child.

Finally, before stopping the replacement therapy and returning to a less intense rhythm of phlebotomy, cryopreservation of spermatozoa may be offered to them.

## Conclusion

Iron overload causes endocrine dysfunction, particularly on the gonadotropic axis, which is responsible for subfertility or infertility. However, the impact of hemochromatosis on reproductive function is multifactorial and depends mainly on pituitary and testicular damage despite the fact that liver or pancreatic damage can also play a role. A decrease in serum testosterone level in hypogonadal patients causes a decline in sexual desire and reduces ejaculation frequency impacting on the quality and quantity of semen. Nearly half of the men affected by hemochromatosis suffer from sexual disorders: erectile and ejaculation dysfunction and libido disorders. Knowledge of the presence of hemochromatosis and its complications in an infertile patient would help the andrologist to determine the best and most rapid strategies to normalize his gonadotropic function and to optimize his fertility.

## Abbreviations

BM: Basal membrane
DMT1: Divalent Metal Transporter 1
ES: Elongated spermatid
FSH: Follicle Stimulating Hormone
GnRH: Gonadotropin Releasing Hormone
hCG: Human Chorionic Gonadotropin
HH: Hypogonadotropic Hypogonadism
IHH: Idiopathic Hereditary Hemochromatosis
LH: Luteinizing Hormone
PAS: Periodic Acid Schiff
PCS: Pachytene spermatocyte
PLS: Preleptotene spermatocyte
RS: Round spermatid
SC: Sertoli cell
SFT: Seminiferous tubule
SG: Spermatogonia
TfR-1: Transferrin Receptor 1
TfR-2: Transferrin Receptor 2
